# Conservative Approach in Managing Complex Odontogenic Lesions: A Case Report and Literature Review

**DOI:** 10.7759/cureus.66056

**Published:** 2024-08-03

**Authors:** RJ Vijayashree, Deepanjali Megarasu, Jeyaseelan Ramasamy, Shubhra C Aramanai

**Affiliations:** 1 Oral and Maxillofacial Pathology, Meenakshi Ammal Dental College and Hospital, Meenakshi Academy of Higher Education and Research, Chennai, IND; 2 Oral and Maxillofacial Surgery, Meenakshi Ammal Dental College and Hospital, Meenakshi Academy of Higher Education and Research, Chennai, IND; 3 Oral Pathology and Microbiology, Meenakshi Ammal Dental College and Hospital, Meenakshi Academy of Higher Education and Research, Chennai, IND

**Keywords:** periapical cyst, decompression technique, conservative approach, radicular cyst, odontogenic cyst

## Abstract

Radicular cysts are among the most common odontogenic cystic lesions in the maxillofacial region. This case report details the management of a large radicular cyst in the anterior maxillary region of a 32-year-old female patient and includes a literature review on such cysts. The patient underwent cyst decompression, surgical enucleation, tooth extractions, root canal treatments, periapical curettage, and prosthetic rehabilitation. This study underscores the effectiveness of conservative approaches, such as decompression, in reducing cyst size and highlights the importance of individualized treatment plans for achieving optimal outcomes and preventing recurrence. Collaborative efforts between clinicians and multidisciplinary teams are crucial for managing radicular cysts and ensuring long-term oral health for patients.

## Introduction

Cystic lesions are among the most common abnormalities encountered in the maxillofacial region. Within this category, different types of odontogenic cysts have been reported, with the radicular cyst being the most prevalent. A radicular cyst, originating from residual epithelial cells in the periodontal ligament following pulp necrosis-induced inflammation, represents a common inflammatory odontogenic cystic lesion in the jaw [1]. Typically situated at tooth apices, they may also occur along root lateral aspects, particularly those associated with accessory root canals. They are typically slow growing and asymptomatic, unless infected secondarily. They are commonly detected incidentally through periapical radiographs of teeth with non-vital pulps. Dental caries represent a common etiological factor, particularly in primary dentition, while traumatic injuries to teeth also contribute to their formation. Their progression varies, with some regressing, remaining stable, or enlarging [2].

Treatment modalities encompass nonsurgical endodontic therapy like root canal treatment of the involved tooth or surgical approaches, with surgical options like marsupialization or enucleation chosen based on lesion characteristics such as size, location, bone integrity, and proximity to vital structures. Nevertheless, conservative management is preferred whenever possible [1]. Anavi et al. [3] found that 60% of the cases showed good ossification of the area following decompression in odontogenic cysts. Additionally, they observed that in 17 cases of radicular cysts, there was a slightly higher reduction in lesion area (85.64%) compared to dentigerous cysts (81.52%) and keratocystic odontogenic cysts (78.85%).

This comprehensive understanding of radicular cysts and their management underscores the importance of tailored treatment strategies to ensure optimal outcomes and prevent recurrence. We present a radicular cyst case treated with a customized conservative approach.

## Case presentation

A 32-year-old female patient presented with a chief complaint of redness over the gums in the upper front tooth region for the past month. She reported a history of redness extending over the previous few months but denied any pain or bleeding during brushing. The patient also disclosed a history of trauma to the upper anterior maxilla during childhood, as well as a root canal treatment and subsequent surgical excision in the upper anterior tooth region six years ago. The past histopathology report indicated an infected dental cyst.

**Figure 1:**
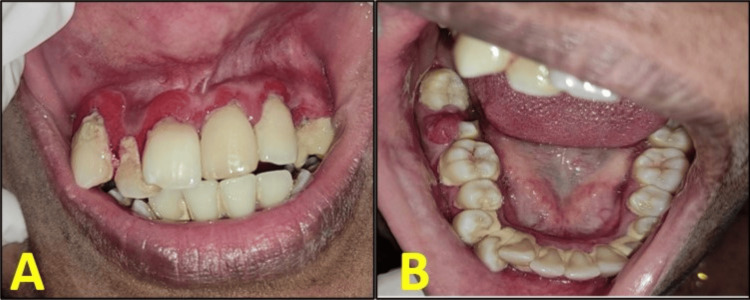
(A, B) Preoperative clinical images of the anterior maxillary region and the occlusal surface of tooth 47 (A) Inflamed and erythematous gingiva in the anterior maxillary region from teeth 13 to 23. (B) Hyperplastic and erythematous gingiva in the region of tooth 47.

Intraoral examination revealed erythematous gingiva over the regions of teeth 13 to 23, with noticeable pus discharge from the gingival sulcus. Teeth 11, 21, and 22 exhibited Grade II mobility, and root stumps were observed in relation to tooth 23. Similar erythematous gingiva was noted in the regions of teeth 43 to 33. Additionally, an irregular exuberant growth was present on the occlusal surface of tooth 47, originating from the pulp chamber (Figure 1A, 1B).

Surprisingly, there was no tenderness on the percussion of any tooth. The vitality test using heat application was negative for teeth 11, 12, 21, and 22. Poor oral hygiene was evident from the accumulation of plaque and calculus. The patient underwent routine blood tests and radiographic investigations.

**Figure 2:**
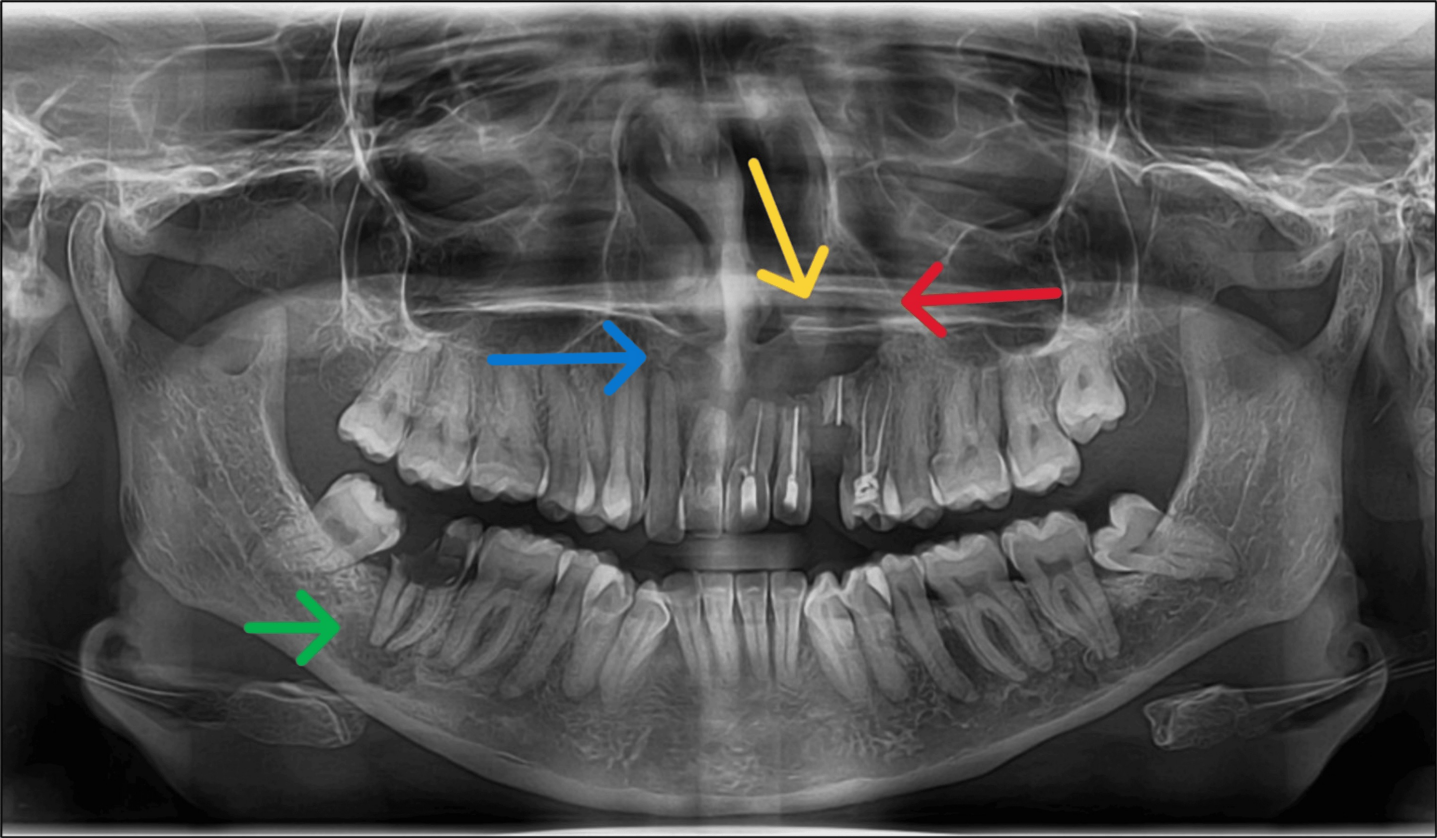
Preoperative orthopantomogram A preoperative orthopantomogram showing an ill-defined radiolucency (blue arrow) extending mediolaterally from teeth 13 to 24. The radiolucency also extends into the maxillary sinus (red arrow) and superoinferiorly into the nasal cavity (yellow arrow). The green arrow indicates radiolucency in the 47-tooth region.

An orthopantomogram revealed an ill-defined radiolucency measuring approximately 2.5 × 2 cm. The lesion extended mediolaterally from the 13 to 24 regions and superoinferiorly into the nasal cavity, causing resorption of the floor of the nasal cavity on the left side. Additionally, there was root resorption observed in relation to teeth 11, 21, 22, and 23 (Figure 2).

Another ill-defined radiolucency was present at the root apex of tooth 47. To confirm the extent of the lesion, a cone beam CT (CBCT) scan was performed.

**Figure 3:**
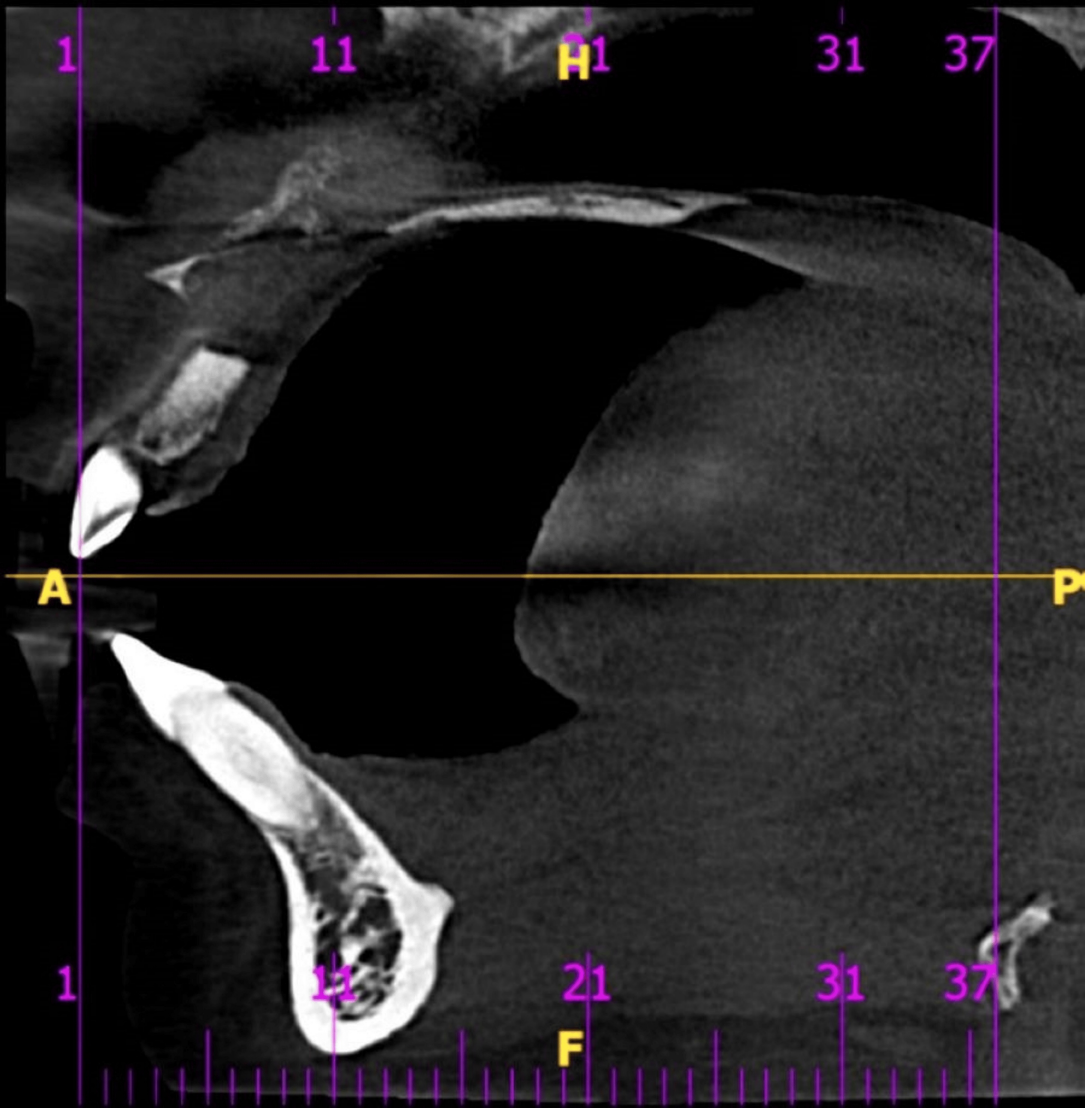
Sagittal section of the CBCT of the anterior maxilla A sagittal section of the CBCT of the anterior maxilla showing a hypodense lesion with resorption of the nasal floor, maxillary anterior roots, and both buccal and palatal cortical bone. CBCT, cone beam CT

CBCT revealed a moderately defined radiolucent lesion measuring 23 × 19 × 9 mm (L × B × W at the greatest dimension). The lesion extended from the alveolar crest in the 21 region to the nasal floor, causing elevation and perforation of the nasal floor. Mediolaterally, it extended from the distal aspect of the 11 to 24 region. Buccopalatally, it caused perforation of the buccal and palatal cortical plates. Root apex resorption of teeth 21, 22, 23, and 24 and involvement of the nasopalatine nerve canal were noted (Figure 3).

An ill-defined periapical radiolucency was evident beneath the roots of 47, measuring 11.5 × 11 × 8.5 mm (L × B × W at the greatest dimension). The lesion extended superior-inferiorly from the alveolar crest to 3 mm above the base of the mandible and mediolaterally around the roots. It caused thinning of the buccal cortical plate and was seen surrounding the inferior alveolar nerve (IAN) canal. Correlating the clinical and radiographic findings, a diagnosis of an infected radicular cyst in the anterior maxillary region and an infected radicular cyst in relation to the 47 region was given.

Initially, cyst decompression was performed in the anterior maxillary region under local anesthesia following the extraction of tooth 23. A stent was then secured using a 3-0 silk suture in the extracted socket area. Additionally, surgical enucleation of the cyst in the right mandibular region was carried out, along with the extraction of tooth 47. All excised tissues were sent for histopathological examination. Postoperatively, the patient was advised to perform intra-socket irrigation, alternating between betadine and chlorhexidine, at least four times per day for a period of three months.

**Figure 4:**
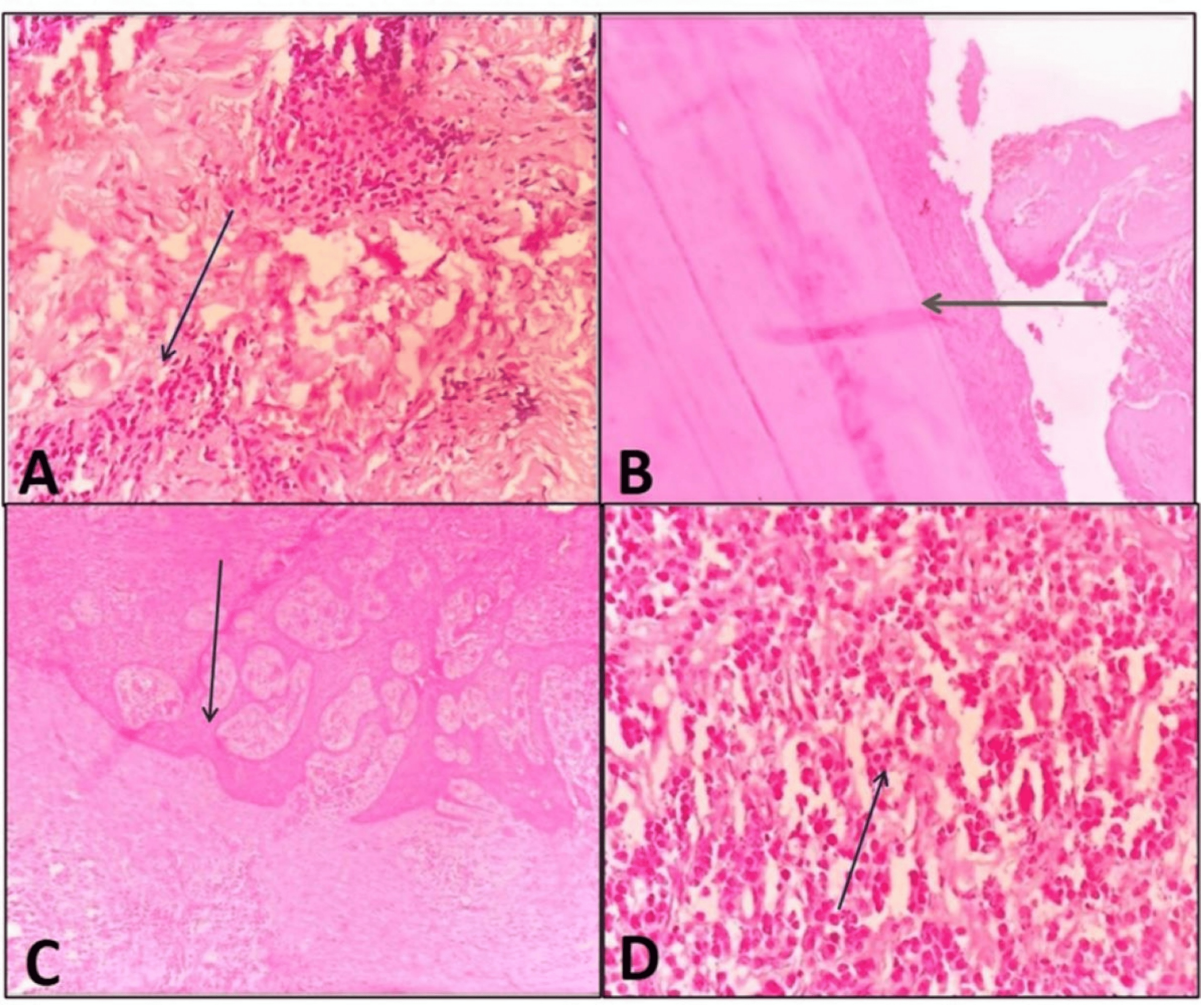
(A-D) Histopathological images of H&E-stained sections at 4×, 10×, and 40× magnifications (A) Fibrous connective tissue with islands of odontogenic epithelium (arrow). (B) Tooth components with adjacent periodontal ligament fibers (arrow). (C) Hyperplastic stratified squamous surface epithelium with an arcading pattern (arrow). (D) Dense mixed inflammatory cell infiltrate, predominantly neutrophils, lymphocytes, and plasma cells.

Histopathological examination of the tissue sections from tooth 23 revealed an odontogenic epithelium composed of non-keratinized stratified squamous cells with a thickness of two to four cell layers. The connective tissue wall exhibited a chronic inflammatory cell infiltrate, moderate vascularity, and extensive areas of hemorrhage (Figure 4A).

Histopathological examination of the mandibular region tissue sections revealed an odontogenic epithelial lining composed of non-keratinized stratified squamous cells of variable thickness. The connective tissue wall exhibited intense chronic inflammatory cell infiltrate, moderate vascularity, and areas of hemorrhage. Additionally, the sections showed evidence of tooth structure, including dentinal tubules and highly vascularized pulp tissue (Figure 4B).

Another section from the same site demonstrated parakeratinized stratified squamous epithelium of variable thickness with areas of pseudoepitheliomatous hyperplasia (Figure 4C). The underlying connective tissue stroma exhibited intense infiltration by mixed inflammatory cells, predominantly lymphocytes and plasma cells, with a few neutrophils. The tissue showed moderate vascularity and areas of hemorrhage (Figure 4D).

Based on the histopathological findings, the final diagnosis was a periapical cyst in relation to the 11-24 region and chronic hyperplastic pulpitis with a periapical cyst in relation to 47.

**Figure 5:**
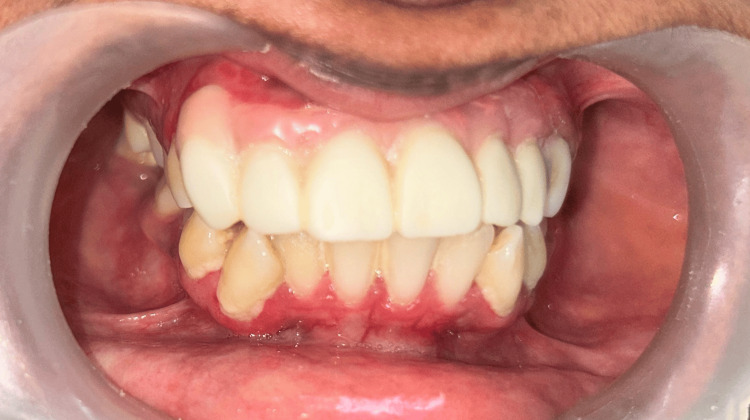
Postoperative clinical image of the anterior maxilla Postoperative clinical image showing the fixed partial prosthetic denture in the anterior maxilla.

Extraction of teeth 22, 21, and 11, along with periapical curettage, was performed three months later due to the lack of improvement in tooth mobility. Root canal treatment for teeth 12, 13, and 25 was completed two weeks afterward. Fixed partial prosthetic rehabilitation was carried out six months later (Figure 5).

**Figure 6:**
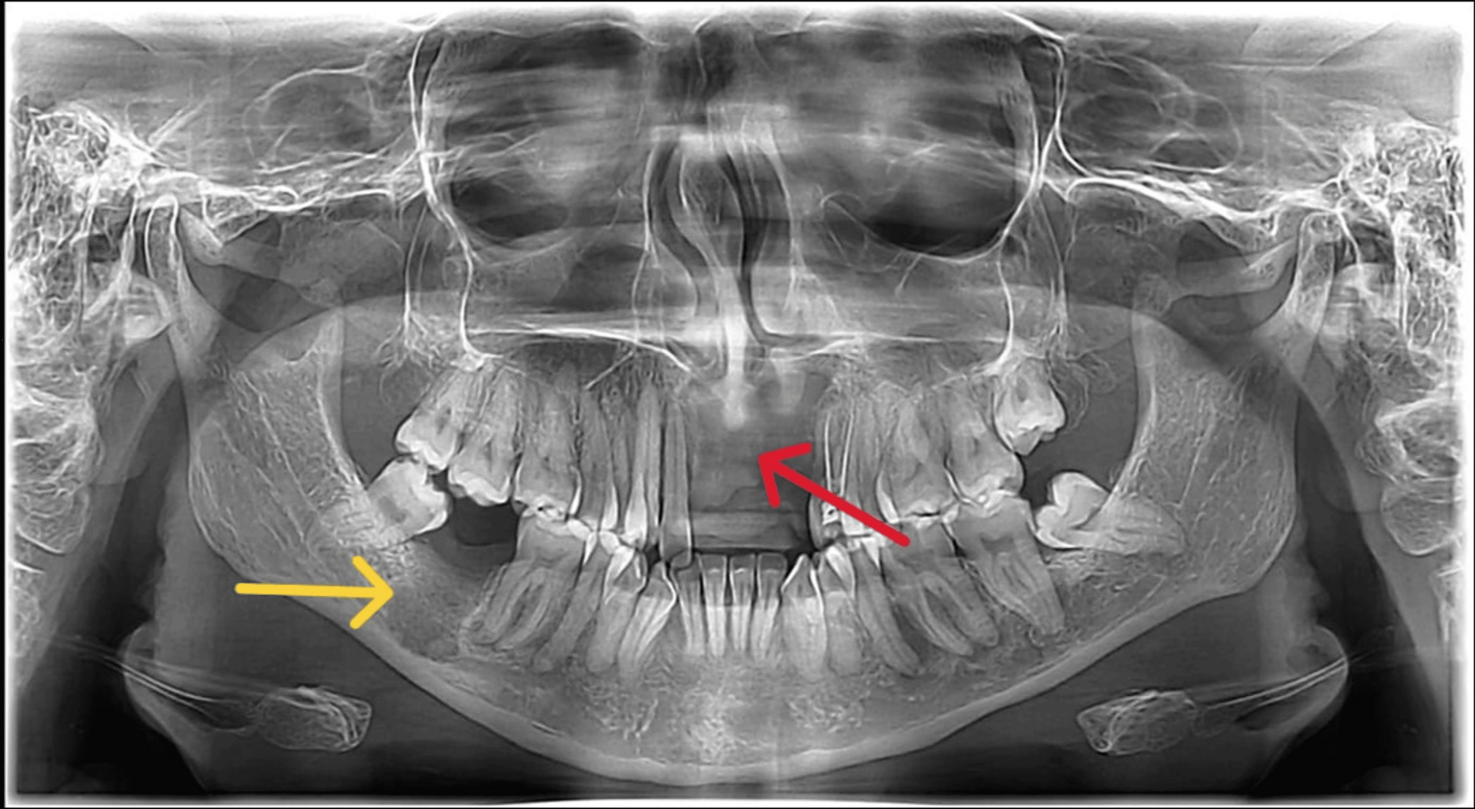
Postoperative orthopantomogram Postoperative orthopantomogram showing bone replacement in the anterior maxillary region (red arrow) and the 47-tooth region (yellow arrow).

Six months after treatment, follow-up revealed good healing and bone formation (Figure 6).

## Discussion

A periapical or radicular cyst is the most frequently encountered cystic lesion in the jaw, with prevalence rates ranging from 6% to 55%. These lesions typically manifest as painless swelling, root resorption, and non-vital teeth. However, in instances of larger lesions, they can affect critical anatomical structures like the maxillary sinus, nasal floor, and IAN [4]. In this present case, the reported patient had large painless lesions with a non-vital tooth-resorbed root and resorption of buccal and palatal cortical bone, as well as involvement of the maxillary sinus, nasal cavity, and nasopalatine canal.

Although invasive surgical methods like enucleation and resection are commonly chosen as the primary treatments [5], they carry risks of serious complications such as facial deformities, maxillary bone fractures, tooth loss, paraesthesia, and recurrence [6,7]. According to Ruslin et al., 3.2% of recurrences were reported following enucleation [8].

Conservative surgical approaches like marsupialization and decompression may be viable alternatives. These techniques are less invasive, and multiple studies have demonstrated favorable outcomes in reducing jaw lesions [9,10]. Thus, it is crucial to consider the patient’s age, lesion location, size, and histological diagnosis to optimize treatment outcomes [7,9]. Since this is a recurrent large lesion with the involvement of adjacent anatomical structures, we have decided to use the tube decompression technique.

The decompression technique entails suturing a device, such as a surgical stent, across the cystic cavity and oral mucosa [11]. This helps diminish the size of cystic lesions by releasing intraluminal pressure, which facilitates gradual bone regeneration. By reducing lesion size, the risk of surgical damage to critical anatomical structures can be minimized [10,11]. The decompression method is also considered the most suitable treatment for children because it preserves the advantageous regenerative potential of both bone and soft tissues in the developing craniofacial skeleton [12].

Kwon et al. conducted a study to assess the three-dimensional volumetric changes of cystic lesions using the decompression method. Their findings revealed a significant reduction of 54.68% in cyst size over an average observation period of 9.41 months. The study concluded that decompression is an effective procedure for reducing cyst size across all patients, as demonstrated by three-dimensional volumetric analysis. Additionally, they observed that decompression yielded even better results when performed over a longer period, in younger patients, and in cases affecting the posterior maxilla [13].

Additionally, this technique carries drawbacks such as the need for long-term intraoperative and postoperative follow-up, challenges in maintaining oral hygiene, and, in certain cases, the requirement for secondary surgery [9]. In our specific case, we applied this technique to treat a recurring large cystic lesion in the anterior maxillary region, achieving notably better outcomes without requiring additional surgery. Similarly, Torres-Lagares et al. reported successfully managing a large inflammatory cyst in the maxilla using the cyst decompression technique. They observed significant peripheral bone formation and separation of the cyst from the maxillary sinus and nasal floor within three months postoperatively [14].

## Conclusions

Radicular cysts are common odontogenic lesions that arise from inflammatory processes secondary to pulp necrosis. Their typically asymptomatic nature and potential for enlargement require careful monitoring and timely intervention. Treatment options vary from conservative approaches to more invasive surgical techniques, with the choice depending on the specific characteristics of the lesion. Marsupialization and enucleation are key surgical interventions, although conservative management is preferred when feasible. A thorough understanding of the etiological factors and clinical behavior of radicular cysts is essential for effective treatment and prevention of recurrence. By adopting a multidisciplinary approach and prioritizing conservative measures where appropriate, clinicians can achieve favorable outcomes and promote long-term oral health for their patients.
